# Does Separating Intentionality From Mental Representation Imply Radical Enactivism?

**DOI:** 10.3389/fpsyg.2018.01497

**Published:** 2018-08-28

**Authors:** Tobias Schlicht

**Affiliations:** Institute of Philosophy II, Ruhr-Universität Bochum, Bochum, Germany

**Keywords:** intentionality, representation, naturalism, autopoiesis, enactivism, cognition

## Abstract

Traditionally, intentionality is regarded as that feature of all and only mental states – paradigmatically beliefs and desires – in virtue of which they are directed at or are about something. The problem of intentionality is to explain how it fits into the natural order given the intuition that no physical entity can be intentionally directed in this sense. The basic assumption of this paper, proposed by enactivists, is that failure to naturalize intentionality and mental representation is partly due to the fact that most participants in the debate take intentionality and mental representation to be equivalent. In contrast, it is proposed to treat intentionality as a feature of whole embodied agents (paradigmatically organisms) who can be directed at objects and states of affairs in various ways, while representation should be regarded as a feature of mental states (and their respective vehicles or underlying mechanisms). The present paper develops and motivates the distinction, applies it to Metzinger’s project of naturalizing phenomenal representation, and demonstrates the range of theoretical options with respect to a delineation of cognition given the enactive proposal. It is taken as problematic that enactivism takes the realm of cognition to be identical to the realm of biology. Instead, a constraint on a theory of intentionality and representation is that it should delineate the subject matter of cognitive science and distinguish it from other sciences, also to leave room for the possibility of artificial intelligence. One important implication of the present proposal is that there can be creatures which can be intentionally directed without having the capacity to represent. That is, their intentionality is restricted to being able to be directed at existent things. Only creatures in possession of the right kind of neurocognitive architecture can produce and sustain representations in order to be directed at non-existent things. It is sketched how this approach conceives of intentionality as a developmental and layered concept, allowing for a hierarchical model of varieties of intentionality, ranging from the basic pursuit of local environmental goals to thoughts about fictional objects.

## Intentionality and Representation in Cognitive Science

What is the most promising approach to cognitive phenomena within a naturalistic framework? Is there a mark of the cognitive, i.e., a feature that all and only cognitive phenomena share? Can all cognitive phenomena be explained by one and the same approach? This paper considers various possibilities of how intentionality, one of the most important features of the mind, can be naturalized, and thus provide a foundation for our most sophisticated form of intentionality, i.e., phenomenal intentionality involving mental representation.

Until recently, cognitive science was based on the undisputed foundation that cognition must be explained in terms of mental representation and computation. For a long time, the dominating theoretical framework of *Cognitivism* conceived of cognition as information processing along the lines of digital computers, in particular as constituted by syntactically driven manipulations of representational structures in the brain that are “sandwiched” (Hurley, 1998) between sensory inputs and motor outputs (e.g., Fodor, 1975; Pylyshyn, 1984). Thus, when I look at the coffee mug in front of me, sensory information hitting my retina is processed in a piecemeal fashion in specialized modules that eventually produce a detailed three-dimensional image of the mug that can in turn guide possible actions like grasping it. The parallel distributed processing movement presented a departure from the simple computer metaphor by modeling cognition using connectionist networks inspired by the architecture of the brain (Smolensky, 1988; Churchland and Sejnowski, 1992). However, even though such artificial neural networks process input subsymbolically, most successful models of this kind still rely on intermediate states which allow for storing, manipulating, and transforming information (about the mug, say) before producing the output (O’Brien and Opie, 2004).

More recently, however, proponents of *Enactive* and *Embodied* approaches to cognition challenged both this representationalist framework and its explicit separation of perception and action in favor of a robustly dynamic approach to cognition. Cognition is now conceived as primarily a bodily activity of a whole organism (or more generally, embodied agent) that can be explained without appeal to mental representations (Varela et al., 1991; Noë, 2004; Chemero, 2009; Froese and Di Paolo, 2011; Hutto and Myin, 2013; Gallagher, 2017). Perceiving the coffee mug not only requires multiple actions like eye-, head-, and body-movements (gaze turning, etc.), perceiving is in the service of detecting action possibilities (like grasping, say) from the start. Enactive accounts come in different varieties, but they all share many assumptions regarding the nature of perception and cognition, apart from rejecting a representational approach, and can be seen as differing mainly with respect to different aspects that are highlighted by them. Proponents of *Sensorimotor Enactivism* emphasize the action-involving character of perception (e.g., Noë, 2004), while the focus of *Autopoietic Enactivism* is on the self-organizing and autopoietic character of basic intentionality; this amounts to claiming a continuity of life and mind – phenomena allegedly sharing the same basic organizational features (e.g., Thompson, 2007). *Radical enactivists*, like Hutto and Myin (2013, 2017), propose to separate intentionality from mental representation and consider basic cognitive capacities as merely intentionally directed without them being representational or content-involving. This is a bold move that introduces a distinction between contentless and content-involving cognition. Although there is much more to be said about all these variants, this brief sketch shall suffice for now, since we will return to some of these claims below.

Finally, proponents of so-called *Predictive Processing* models of the brain claim to have found “the first truly unifying account of perception, cognition and action” (Clark, 2016, p. 2) by conceiving of the brain as a prediction machine, which is constantly testing hypotheses about the incoming sensory stimulation based on a hierarchical generative model that is constantly updated based on prediction errors signaled by forward neural processing (Friston, 2010; Hohwy, 2013; Metzinger and Wiese, 2016). On this view, when I perceive the mug, brain processes have already formed a set of top-down expectations or predictions about the incoming sensory information based on the most likely hypothesis given prior knowledge about certain parameters of the situation. These expectations are then matched against the actual sensory input resulting in bottom-up neural signals. Deviations from the prediction constitute prediction errors and result in the relevant update of the brain’s generative model of the situation. The overarching goal (and unifying principle) of the brain’s perceptual and cognitive activities is to minimize prediction error. Given the promise that the Bayesian prediction machine hypothesis could yield a unifying framework for cognition, it is not surprising that both representationalists and enactivists engage in a hot debate regarding whether this framework should be interpreted in representationalist (Hohwy, 2013; Clark, 2015) or enactivist terms (Hutto and Myin, 2017).

These roughly three frameworks portray cognitive phenomena quite differently since they suggest explanations using different explanatory tools. One of the central questions that this debate gives rise to is whether cognition is in general representational or content-involving and whether any adequate theory of cognitive phenomena must invoke mental representations. It has been an almost undisputed assumption in cognitive science that intentionality and representation can be used synonymously (or are at least equivalent). Most publications on naturalizing intentionality have thus used these terms interchangeably. To give two random examples, Crane (2003, p. 30) says explicitly that “philosophers have a word for the representational nature of states of mind: the call it ‘intentionality’.” Similarly, Searle (1983, p. 4) holds that “intentional states represent objects and states of affairs.” Since representations have content, they also have accuracy conditions. By contrast, echoing points made by Dreyfus (2002), Hutto and Myin (2017, p. 95) propose a “radical enactivism,” according to which we should “think of the most primitive form of intentionality […] in non-contentful, non-representational ways” but still as an “attitude directed toward an object.” Intentionality is then no longer a feature of contentful mental states that represent but “an attitude of the whole organism expressed in their behavior” (Hutto, 2008, p. 57). The idea is to disambiguate Brentano’s initial characterization of intentionality as *directedness* and *aboutness*, reserving the latter for those forms of cognition that depend on socio-cultural capacities and cultural symbol-systems, such that contentful representations with satisfaction conditions only appear on the scene in the wake of linguistic capacities.^1^ This move leads Hutto and Myin to the even more radical claim, however, that all basic cognitive capacities, in particular perception and action, but also forms of imagination and memory, can be exhaustively explained without the assumption of mental representations and content.

Thus, one could express this radical departure from the tradition by formulating a set of theses that express the traditional view in cognitive science, i.e., the *Equivalence Thesis* and the *Separation Thesis*:

### Equivalence Thesis (ET)

Intentionality and mental representation are equivalent and co-extensive notions referring to the directedness or aboutness of mental states.

### Separation Thesis (ST)

Intentionality and representation can be investigated and accounted for independently of consciousness.

The separation thesis is not a target of this paper, although it is part and parcel of classic cognitive science until today. In line with proponents of Radical Enactivism, the present paper approaches the naturalization of intentionality by rejecting the Equivalence Thesis which can be partly blamed for lack of progress and agreement in this area. As will become clear in a moment, this crucial conceptual move opens up new avenues to the naturalization of intentionality *independently* of the naturalization of mental representation. But this paper considers a systematic position that is only related, yet importantly different from Radical Enactivism. Intentionality and representation should be treated separately, in agreement with Radical Enactivism. But given that it is questionable whether Radical Enactivism can yield a promising and useful framework for cognitive science, it will be explored whether their rejection of mental representation and content may be the only way of dealing with the separation of intentionality and mental representation. In his review of Hutto and Myin’s recent book, Thompson (2018) observes that “they insist that content entails correctness conditions. They also apparently think that only representations can have correctness conditions. So, any form of intentionality (cognitive directedness) that lacks correctness conditions or is not representational is said to be contentless.” By considering a phenomenological notion of content that is not propositional and thus does not imply correctness conditions, Thompson points at alternatives neither explored nor discussed by Hutto and Myin and other radical enactivists. By pointing at some shortcomings of the radical enactive proposal, this paper discusses different systematic alternatives that consider other routes after separating intentionality from mental representation.

The guiding questions of this paper have been formulated at the outset: They concern the task of identifying a mark of the cognitive. The core intuition supporting the rejection of the Equivalence Thesis is that while intentionality (as directedness) should be conceived as a feature of whole embodied agents, mental representation is a feature of the mechanisms sustaining cognitive states and processes. Consequently, the reason why intentionality and representation should be distinguished is not that (basic) cognition is non-representational, as enactivists would have it. The reason is that these different notions denote features of different entities. It is organisms or agents, not mental states that are directed. And it is mental states (and their underlying mechanisms), not organisms or agents, which represent. Consequently, an organism or agent can be intentionally directed at something without representing it. And if an organism is in possession of mechanisms (and thus mental states and processes) that can represent, then this can enhance its range of possibilities to be directed at objects and states of affairs. For example, representational mechanisms can enable an organism to be directed at fictional objects via the imagination. But importantly, there can be simple creatures that are intentionally directed at something without thereby representing it. This proposal must be spelled out in more detail but it gives rise to a set of further interesting questions that should be explored: For example, (1) Is non-representational intentionality cognitive or not? It may well be that cognition merely constitutes a special case of intentionality that is enabled by mental representations. Then the range of intentionality would be bigger than the range of cognitive phenomena. This would be in stark contrast to the thesis of Radical Enactivism. Choosing this option would leave mental representation as a candidate for delineating the cognitive realm and avoid the consequence that all forms of intentional directedness in nature would have to be considered as cognitive. (2) If non-representational intentionality constitutes a form or forms of cognition after all, then what is the mark of the cognitive and how do these forms of cognition relate to the more sophisticated forms that involve mental representation? (3) How does the most sophisticated form of intentionality of conscious thought, i.e., the one that is phenomenally experienced and produces intensional contexts, related to basic intentionality that is neither phenomenal nor representational? Does it delineate a special domain within the realm of the cognitive? Or is it rather the source of all intentionality, as various philosophers, though rarely any cognitive scientists, maintain (e.g., Searle, 1992; Pitt, 2004; Strawson, 2004; Mendelovici, 2018)?

Given the thematic focus of this Special Issue, this paper situates the present discussion in relation to Thomas Metzinger’s work in order to demonstrate options for providing a naturalistic foundation for his theory of a subclass of mental representation, namely *phenomenal representation*. The rest of the paper proceeds as follows: By way of setting the stage, the following Section “Phenomenal Representation” outlines Metzinger’s approach to representation and mental representation and highlights those points where the present conceptual proposal can usefully complement his account. Section “The Homeostatic Basis of Phenomenal Subjectivity” then turns to a discussion of the role of the organism’s body and central nervous system for sustaining phenomenal representation, while Section “Self-Organization and Basic Biological Intentionality” sketches how this naturalistic grounding of phenomenal representation in the body can be extended to a more general naturalistic account of intentionality (that is independent of mental representation) by relying on enactivist ideas, while discussing conceptual routes that are quite different from enactivism.

## Phenomenal Representation

Many philosophers of mind take it for granted, indeed to be an “obvious fact that biological nervous systems are able to generate representations of the world and its causal matrix by forming internal states which then function as internal representations of this causal matrix” (Metzinger, 2003, p. 18). This is the attitude, expressed paradigmatically by Searle (1992), that biological phenomena cause mental phenomena. Functionalists do not question this claim, but typically take the more liberal attitude of allowing for artificial systems to be capable of generating the same types of mental phenomena using other means, downplaying the biological details needed for causing mental phenomena. In his monumental work *Being No One*, Metzinger’s (2003) project is not to demonstrate how intentionality *tout court* can be naturalized. He explicitly states that “intentionality as such is not an epistemic target” of his representationalist approach to the first-person perspective. He develops his theory of subjectivity in terms of a phenomenal self-model and a phenomenal model of the intentionality relation, and this theory takes for granted that organisms are capable of representing. It is explicitly not his “goal to offer a general theory of mental representation” (Metzinger, 2003, p. 595). Therefore, for his theory to be thoroughly naturalistic, it presupposes *some* naturalistic explanation of representation and intentionality in general, while his goal is merely to demonstrate how his theory can account for the *phenomenal experience* of intentionality, i.e., phenomenal content. His crucial philosophical step “consists in *phenomenalizing intentionality”* which may be a “necessary detour […] in the project of *naturalizing* intentionality *tout court.*” (Metzinger, 2003, p. 414) Thus, his restricted claim is that

“the *phenomenal* experience of being an intentional agent, of being a perceiving, attending, and cognizing subject, can be naturalized. Of course, this in no way precludes the possibility that intentional content *as such* can never, and maybe even for principled reasons, be naturalized. But getting the first obstacle out of the way may greatly help in gaining fresh access to intentionality *as such* […]. We can separate the issue of consciously experienced intentionality from the more general problem of how something like representational content could evolve in the minds of human beings and other animals *at all.*” (Metzinger, 2003, p. 414)

Note that the last part of this quote indicates an acceptance of both the Equivalence Thesis and the Separation Thesis, since he argues that conscious or phenomenal intentionality is to be separated from intentionality as such which is then identified with representational content. Thus, the aim of his theory is to address the question how the consciously experienced arrow of intentionality can be explained in terms of a transparent model of the intentionality relation, i.e., a model that cannot itself be recognized by the subject *as* a model. And although he does not provide a naturalistic theory of intentionality as such, he nevertheless speculates that “the ‘real’ intentionality relation” may be “constituted by an active cognitive agent interacting with its environment” (Metzinger, 2003, p. 113). Indeed, he considers the possibility that “intentionality” may perhaps be anchored “[…] on a prerational level, probably starting with the motor system and early levels of attentional processing […]” (Metzinger, 2003, p. 414). These brief comments suggest that Metzinger could agree in principle with some enactivist claims, e.g., the importance of an embodied agent interacting in various ways with their environment in order to constitute an intentionality relation. Indeed, his acceptance of the importance of the aspect of embodiment is emphasized in Metzinger (2014).

Since Metzinger (2003, p. 415) is “mute about the question whether anything like ‘real’ intentionality exists” or what a naturalistic explanation of it looks like, it is the aim of this present paper to use the proposed broader framework in order to take some steps in the direction of a naturalistic grounding of his theory of phenomenal intentionality. Metzinger (2003, p. 15) is a friend of useful distinctions: As he uses the term, “mental representation is a process by which some biosystems generate an internal depiction of parts of reality.” Such internal representations can be experienced and used by the respective system in order to guide its flexible behavior. In contrast to genuinely mental representations, lots of information processing that has to do with the regulation of heart rate or immune system parameters, can “certainly carry information, but this information is not *mental* information” (Metzinger, 2003, p. 17). That is, although it *tracks* states of affairs pertaining to the internal milieu of the body and thus *concerns* the organism, it may only *affect* mental phenomena but it is not already identical to the mental phenomena in question. According to Metzinger, such representations do not count as mental representations because they cannot become conscious. They are internal representations in a “purely physical sense” (ibid.). In contrast, mental representations “can, at least in principle, possess a phenomenal kind of ‘inwardness”’ (Metzinger, 2003, p. 18). Thus, Metzinger distinguishes, importantly, between *representations* and *mental representations*, while he stipulates that the latter are separated from the former by their *phenomenal* features. Arguably, if the fact that heart rate and the immune system carry information is sufficient for them to count as representations, then representations abound in nature. Even the infamous tree rings, which carry (or embody) information about the age of the tree, then count as representations. But *mental* representations do not permeate nature so that we need to draw distinctions anyway. The question is where to draw the line and which criteria we should use to draw explanatorily useful boundaries. The relationship between intentionality and mental representation is thus complicated by the fact that *mental* representations only form a subclass of representations in general, whether or not we take *phenomenality* to be the correct criterion or not. Given that *representations*, merely understood as entities indicating information, (Dretske, 1981; Rupert, 2018), seem to abound in nature and are thus insufficient to characterize cognition, the question arises whether intentionality at least coincides with representation if not with mental representation. The conceptual move proposed in this paper implies the following tasks: (1) We must characterize intentionality independently of representation and demonstrate how it can be conceived as a natural phenomenon. (2) We must provide criteria for distinguishing (mere) representation from genuine *mental* representation in order to capture the most distinctive human mental capacities like conscious thought, imagination and so on. Of course, this twofold task cannot be achieved in this single paper. But it is an important task since it forces us to delineate the cognitive from the non-cognitive realm. Identifying a mark of the cognitive is no trivial task but it has important ramifications for cognitive science and its subject matter, as we will see below.

In order to supplement Metzinger’s approach to phenomenal representation by providing a naturalistic theory of intentionality and representation, the first thing to do is to develop a naturalistic theory of intentionality as such. In this vein, the present paper applies the enactivist proposal of separating intentionality from representation and mental representation to this effect, yielding a much more differentiated framework. In addition to Metzinger’s distinction between representation and mental representation, the central claim that mental representation is a feature of mental states, while intentionality is a feature of whole embodied agents – be they natural (organisms) or artificial (robots) – yields the further important distinction between intentionality and representation. As mentioned at the outset, separating intentionality from representation in this way borrows from the recent development of Enactivism, yet, by retaining the notion of mental representation for cognitive phenomena, it differs from this radical position.

## The Homeostatic Basis of Phenomenal Subjectivity

I have quoted quite extensively from Metzinger’s major book *Being No One* above, partly because these quote illustrate how he takes intentionality and mental representation to be equivalent, while considering kinds of representation that are not mental. Interestingly, Metzinger seems to reserve the category of mental representations for those that can in principle be phenomenal. This is in line with Searle’s (1992) Connection Principle, which considers only those representations as mental that can potentially become conscious. Note that any attempt to explain *conscious intentionality* in terms of a representational theory presupposes accepting the separation thesis since if representation wasn’t taken to be independent of consciousness, then this project of formulating a representationalist theory of consciousness would be doomed to fail. Thus, Metzinger must subscribe to the Separation Thesis. Likewise, the quote from the foregoing section demonstrate that Metzinger also subscribes to the Equivalence Thesis. He often uses intentionality and mental representation interchangeably, for example, when he speaks of ‘intentional content’ and suggests that his approach could yield progress regarding the task of explaining ‘intentionality as such’ which he seems to identify – in the same sentence – with the task of showing how ‘representational content’ could evolve in animals.

Metzinger draws heavily on the work of Damasio (1999, 2011) in his naturalistic account of phenomenal subjectivity. This is noteworthy since Damasio’s reliance on the organism’s body, homeostasis and processes of self-organization is a specific instance of a more general approach to the origins of intentionality to be outlined in the following section. Like Metzinger, Damasio is concerned with the sense of self, i.e., the subjectivity of conscious experience. While Metzinger approaches this issue from a conceptual point of view by developing his theory of self-models, Damasio is interested in supplying the underlying machinery responsible for generating and sustaining subjectivity. Thus, their approaches can mutually complement each other since, as Metzinger puts it, the common target is how an organism can

“feel itself as itself. As a physical system continuously engaged in the process of self-organization and self-regulation, the organism has to maintain a robust functional boundary with its environment and keep a large number of internal parameters stable and invariant. A large section of self-presentational content precisely generates a continuous flow of information about the degree of invariance that is currently being achieved.” (Metzinger, 2003, p. 344)

As far as the stability and invariance that a conscious subject experiences are concerned, the body is the obvious candidate for providing the conditions under which a stable reference point of conscious experiences can be provided and sustained. To this effect, Metzinger praises Damasio’s theory of consciousness and self since it highlights “the way in which this flow is very likely rooted in elementary bioregulatory processes, those processes concerned with keeping the internal chemical milieu of the body stable in a continuously changing environment.” (Metzinger, 2003, p. 345)

All intentionality involves a self-other distinction, since understood as directedness it implies that something reaches beyond itself, transcends itself. As far as Metzinger’s target is concerned, the subjectivity of phenomenal representation, Damasio argues that the experienced, yet elusive sense of self is grounded in a biological process realized by a neural mechanism distributed over a cluster of connected brain structures (Damasio, 2011, p. 8f). These biological processes are responsible for the regulation of the whole organism’s wellbeing. In order to maintain its identity and to ensure survival, the organism’s overall homeostatic state must remain within certain bounds. One of the brain’s chief tasks is to monitor and regulate bodily processes on the basis of information that it receives from the body at any given point in time. Because of their monitoring and regulating function with respect to this goal, Damasio calls these brain structures the unconscious biological ‘proto-self.’ This is the analog of Metzinger’s notion of “mental or merely internal (i.e., necessarily non-phenomenal) self-presentation” (Metzinger, 2003, p. 345). For Damasio, it is one of the most important ideas of his framework “that the body is a foundation of the conscious mind” in the sense that these “proto-self structures are not merely *about* the body. They are literally and inextricably *attached* to the body.” That is, although Damasio’s work is mainly concerned with determining the *brain structures* sustaining subjective consciousness, these structures only possess the function that they have in virtue of the body and receiving feedback from the body. They are only considered as representing the body because they *concern* the body. In his framework, these structures ultimately give rise to the phenomenally experienced sense of self, the ‘core self.’ In his attempt to provide such a biological grounding for the sense of self, Damasio (2011, p. 48) emphasizes the connection between “organisms,” “purposes” or “biological needs,” and “value.” Since all organisms must maintain their physiological state “within an optimal homeostatic range,” “management operations” such as “procuring energy, incorporating and transforming energy products…aim at maintaining the chemical parameters of a body’s interior (its internal milieu) within the magic range compatible with life.” This homeostatic process of maintaining the right chemical balance within the body is tied to the organism’s need (or goal) of survival. This notion of *need* is in turn tied to the notion of a *biological value*:

“I see value as indelibly tied to need, and need as tied to life. The valuations we establish in everyday social and cultural activities have a direct or indirect connection with homeostasis. That connection explains why human brain circuitry has been so extravagantly dedicated to the prediction and detection of gains and losses, not to mention the promotion of gains and the fear of losses.” (Damasio, 2011, p. 47f)

In this passage, Damasio suggests a direct lineage starting from the most basic intentional activities of the simplest living creatures to our social and cultural activities, connected via the relation between value, (biological) needs, and life, over various stages of complexity. Dependent on the complexity of the organism, different values are assigned to its physical environment. In organisms with brains, such as human beings, the homeostatic process is monitored and regulated by neurons, peculiar kinds of cells that possess the ability to influence other cells, based on inputs that inform the neurons about the state of the body at all times. If necessary, neurons initiate the release of chemical molecules to reestablish the homeostatic balance. Damasio interprets this in representational terms:

“They (the neurons) end up *representing* the state of the body, literally mapping the body for which they work and constituting a sort of virtual surrogate of it, a neural double […] In brief, neurons are *about* the body, and this ‘aboutness,’ this relentless pointing to the body, is the defining trait of neurons, neuron circuits, and brains.” (Damasio, 2011, p. 38f)

According to Damasio, neurons and the bodily state that they represent also play an important role in our directedness toward – and representation of – the outside world and its objects.

“[…] when the brain maps the world external to the body, it does so thanks to the mediation of the body. When the body interacts with its environment, changes occur in the body’s sensory organs, such as the eyes, ears and skin; the brain maps those changes, and thus the world outside the body indirectly acquires some form of representation within the brain.” (Damasio, 2011, p. 39)

All this suggests that at least organisms with brains and central nervous systems are capable of producing representations and, more specifically, mental representations, where the latter form the subgroup of phenomenally experienced representations, in Metzinger’s terminology.^2^ Moreover, what we have arrived at is a story that indicates the importance of the organism’s body, indeed of the whole organism, for mental phenomena. But both Metzinger’s and Damasio’s accounts are focused on the question which features of the brain constitute the basis of our *phenomenal* sense of self. I agree with Metzinger’s assessment that this rough story, indicated by Damasio, makes a lot of sense. And given that it points toward claims that are associated with proponents of embodied cognition, this story also raises further questions: For example, given that Metzinger and Damasio focus on sophisticated mental phenomena, what about a naturalistic account of intentionality and representation as such? Do all organisms exhibit intentionality and why? And does this mean, given the equivalence thesis, that all organisms represent in virtue of exhibiting intentionality? If not, then where and why should we draw boundaries? Can we formulate criteria that inform us about which instances of intentionality should count as cases of cognition? Thus, the question I want to address now is how we can make headway toward providing this general naturalistic account, extending Damasio’s theory by relying on Autopoietic Enactivism.

## Self-Organization and Basic Biological Intentionality

Brentano (1874/1995) famously claimed that intentionality is the mark of the mental: All and only mental phenomena are intentional, no physical phenomenon exhibits intentionality. This gave rise to the project of naturalizing intentionality, i.e., of demonstrating that Brentano was wrong in thinking that no (broadly) physical entity can be intentional. Fodor (1987, p. 97) famously thought that this would have to be a reductive project: He suggests that once physicists will have completed the “catalog of the ultimate and irreducible properties of things,” then “the likes of *spin*, *charm*, and *charge* will perhaps appear on their list. But […] intentionality […] doesn’t go that deep […] If aboutness is real, it must be really something else.” Again, note that Fodor takes intentionality and mental representation, paraphrased as *aboutness*, to be equivalent here. But explaining intentionality in naturalistically acceptable terms, i.e., “in non-intentional, non-semantical, non-teleological, and in general, non-question-begging vocabulary” (Fodor, 1987, p. 126) turned out to be a rather difficult project, and we have not reached anything like a consensus as to how to fit the phenomena under the umbrella term “intentionality” in a naturalistic view.

The move of separating intentionality from mental representation in a way parallels the one proposed by Metzinger. But rather than singling out a significant case of representation – conscious *phenomenal* mental representation – like Metzinger does, the present paper highlights a distinction between a property that applies on the level of whole embodied agents – intentionality – and a property that applies on the level of individual mental states (and their realizing mechanisms) – representation. While there can be intentionality without mental representation, most likely in simple creatures, those agents which *are* in possession of representational mechanisms will enjoy a much broader range of possibilities to be *directed* at objects and states of affairs. Most importantly, such agents^3^ may be directed at fictional objects like centaurs or Santa Claus, because of their capacities of thought and imagination which are (most likely) realized by representational mechanisms in the brain. Simpler creatures lacking these mechanisms and capacities may be restricted in the sense of only being capable to be directed at things that exist. Whether this is the case depends on the criteria we formulate for such sophisticated directedness, i.e., the directedness that many philosophers take Brentano (1874/1995) to have been concerned with in his original formulation of the intentionality thesis. For example, Morgan and Piccinini (2017) discuss the inadequacy of so-called tracking theories to explain the “distinctively mentalistic phenomenon of directedness toward entities that may not exist that poses the central puzzle of intentionality.” Tracking theories typically posit some tracking relation in virtue of which some mechanism picks out – or represents – a feature or object in the world. Against this move, Morgan and Piccinini (2017) argue that to explain directedness toward a centaur it is insufficient to “posit neurons whose function is tracking centaurs.” Such neurons would in fact never fulfill their function since there are no such things and it is difficult to justify why a mechanism with that function should have evolved. Among the entities that exhibit intentionality, we must formulate criteria in order to delineate the set of entities that are capable of this sophisticated kind of directedness. One route may be to require a certain architecture or functional organization which in turn can only be supplied by certain networks like nervous systems (Rupert, 2018). But there are obviously alternative routes since this is ultimately an empirical issue. Sadly, this issue is also beyond the limited scope of this paper which will focus on the other end of the scale, namely the origins of intentionality.

Damasio seems to suggest that abstracting from the paradigm case of a human being, it is only a question of scale to accept a single cell as the bearer of intentionality, since the cell shares the same basic organization as a complex organism such as a human being. Damasio points to these structural analogies between complex organisms such as human beings and simple organisms such as cells:

“In many respects a single cell is a preview of what a single organism such as ours would come to be. One can see it as a sort of cartooned abstraction of what we are. The cytoskeleton is the scaffolding frame of the body proper, just as the bone skeleton is in all of us. The cytoplasm corresponds to the interior of the body proper with all its organs. The nucleus is the equivalent of the brain. The cell membrane is the equivalent of the skin. Some of these cells even have the equivalent of limbs, cilia, whose concerted movements allow them to swim.” (Damasio, 1999, p. 33)

But the problem is that if one accepts the Equivalence Thesis, like Metzinger and many others, then ascribing intentionality to single cells forces us to also grant that they are capable of representing. Whether they also possess the capacity of mental, i.e., phenomenal representation, would be a further issue. By contrast, rejecting the Equivalence Thesis allows for an ascription of intentionality without granting representational capacities. The main point of this section is that if one accepts, following *all* enactivists, that autopoiesis (self-organization and self-production) is the defining characteristic of organisms and if one is willing to apply intentionality to whole organisms, it would be arbitrary to accept its application with respect to humans and other higher animals but not to cells and plants, given that they are all organisms.^4^ One can already envisage questions concerning the commonalities and differences between putative cognitive capacities in cells, plants, animals, and humans. We will return to this below.

### Autopoiesis and Nano-Intentionality

Some philosophers have suggested that tracing back the natural origins of intentionality may not lead us *all* the way down to the level of physical particles (*pace* Fodor), but at least to the level of biological self-organization. Maturana and Varela (1980) introduced the idea that living organisms are defined by the feature of self-organization or “autopoiesis.” Weber and Varela (2002) refer explicitly to Kant’s (1790/1998) discussion of organisms as natural purposes and the continuing discussion by Jonas (1966). The advantage of this view is that it yields, according to Thompson’s (2007, p. 159) Autopoietic Enactivism, “an explicit hypothesis about the natural roots of intentionality: On this conception, intentionality arises from the operational closure and interactive dynamics of autopoiesis,” and is thus grounded in the structural organization and biological autonomy of living organisms, namely self-organization. Intentionality is conceived as a basic feature of an organism’s embodied interactions with the environment, not as a feature of mental states (ibid., 25). This view can be seen as a descendant, indeed a naturalized version, of Kant’s definition of an organism as a “natural purpose,” developed in his *Critique of the Power of Judgment* (Kant, 1790/1998, pp. 65–66). A natural purpose is a system in which the parts of the system (1) are only possible through their relation to the system as a whole, and in which (2) the parts of the system, moreover, are “combined into a whole by being reciprocally the cause and effect of their form.” That is, “in such a product of nature each part is conceived as if it exists only *through* all the others, thus as if it exists *for the sake of the others* and *on account of* the whole, i.e., as an instrument (an organ) […]” (Kant, 1790/1998, p. 373). Unlike an artifact, such as a watch, an organism is – simply in virtue of being a natural purpose – not caused by any external rational agent, such as a watchmaker, but by its own formative powers. A popular example illustrating the peculiar feature of self-organization characteristic of organisms – alluded to by Kant – is an organism’s ability to repair itself in response to damage to the body. At the time, Kant seems to have been aware of Abraham Trembley’s discovery that after cutting them in two halves, hydra – which are multicellular organisms found in unpolluted fresh waters – regenerate by developing two complete organisms. As Fitch (2008) observes, many organisms like salamanders and zebra fish can regrow entire body parts like lost limbs. This astonishing fact raises the question how it is possible for these animals (or their parts anyway) to “know” what they should grow, i.e., what the overall animal is supposed to be like in order to supplement what’s left with what’s missing. Thompson dubs this process “circular causality,” a combination of *local-to-global determination* whereby emergent structures and properties on the macro-level are generated and sustained by the behavior of the components on the micro-level, and “*global-to-local determination* whereby global structures and processes constrain local interactions” (Thompson, 2007, p. 62). He discusses it in the context of neurodynamics:

“Coherent and ordered global behaviors, which are described by collective variables or order parameters, constrain or govern the behavior of the individual components, entraining them so that they no longer have the same behavioral alternatives open to them as they would if they were not interdependently woven into the coherent and ordered global pattern. At the same time, the behavior of the components generates and sustains the global order. This two-sided or double determination is known as circular causality […]” (Thompson, 2007, p. 62)

Understood in this way, teleology or circular causality is not opposed to causality but introduces a differentiation into the notion of causation in terms of a two-sided dependency. Kant’s notion of a natural purpose delineates the group of those entities which are at the same time products of nature and which necessarily have to be understood teleologically, as being intrinsically directed toward some purpose or goal. Varela recognized that his notion of autopoiesis has an important precursor in Kant’s original discussion of self-organization (Weber and Varela, 2002). But while Kant held teleological descriptions as providing merely an indispensable heuristics rather than an objective explanation (Kant, 1790/1998, p. 389), Varela and Thompson both argue that a modern empirical theory of life (based on the theory of autopoiesis and dynamical systems theory) can be seen as a naturalized version of Kant’s notion of a natural purpose, providing us with a “non-reductionist yet ‘hard’ explanation of the living” (Weber and Varela, 2002, p. 102). According to this modern understanding, the organism is conceived of as a “creator of ‘real teleology”’: […] *organisms are subjects having purposes according to values encountered in the making of their living*” (ibid.). In the present context of the elaboration of the basic biological intentionality of organisms, it is crucial that such biosystems are in an important sense autonomous, i.e.,

“a cell or multicellular organism is not merely self-maintaining, like a candle flame; it is also self-producing and thus produces its own self-maintaining processes, including an active topological boundary that demarcates inside from outside and actively regulates interaction with the environment” (Thompson, 2007, p. 64, cf. 103 for the defining elements of autopoietic systems).

This organization can be illustrated by a look at the simplest organism, the living cell, out of which all complex organisms are ultimately composed. The cell also serves as a model in Varela and Maturana’s initial arguments for the notion of the autopoietic organization that defines organisms: in a single cell, a biochemical network “produces the metabolites that constitute both the network itself and the membrane that permits the network’s bounded dynamics” (Thompson, 2007, p. 65). So, in the first place, a cell qua self-organized and self-producing system generates a simple biological self-world distinction. By itself, this is still insufficient to count as intentionality. But in order to survive and maintain its identity, the cell must continually exchange matter and energy with its environment. Some molecules are imported through the membrane and participate in processes inside the cell, whereas other molecules are excreted as waste. In this way, the cell produces its own components including its boundary, which in turn produce and maintain it as a unified system, in an ongoing process. Autopoiesis is the term to describe this continual self-production (Thompson, 2007, p. 97ff). This brings us back to the discussion of Damasio’s account. In order to sustain itself, the cell must realize biological purposes. With respect to the cell’s biological needs, its physical environment thus obtains a certain *value*. For the cell, features of the physicochemical environment “turn into” nutrition; but only in relation to the cell’s metabolism do they acquire the status of food. In this way, by being an entity with an identity that depends on its environment for survival, the cell is *directed* toward and dependent on its environment, i.e., the relation between cell and environment is asymmetric. By being semi-autonomous, the cell as organism bestows significance and value to the relevant features in the environment. Thus, living itself is a way of bringing forth value and significance. “In this way, the environment becomes a place of valence, of attraction and repulsion, approach or escape.” (Thompson, 2007, p. 158) Accordingly, the semipermeable boundary that enables the cell to exchange matter and energy with its environment can be seen as the “natural root of intentionality: Intentionality arises from the operational closure and interactive dynamics of autopoiesis” (Thompson, 2007, p. 159).

Thompson (2007, p. 159) also illustrates his view by saying that “intentionality first emerges in nature in the form of autopoiesis and sense-making.” To call what the cell is doing here, namely its intentional directedness toward its environment based on its biological needs, “sense-making” suggests that it already exhibits a basic kind of cognition. But there is a danger here that slipping from this notion of biological intentionality to the notions of attitudes and sense-making so quickly blurs rather than illuminates this otherwise persuasive naturalization of intentionality. Thompson (2007, p. 127) is cautious not to take a stand in this matter, but Hutto and Myin (2013, pp. 32–36) object to this suggestive way of describing it. The important point to consider at this juncture is that once we have acknowledged the naturalization of the notion of intentionality (as directedness) provided by Autopoietic Enactivism, various theoretical options how to proceed from here are still available. These options concern not only explanations and restrictions on the notion of cognition, but also specifications of the relations between intentionality, cognition, and representation. The crucial question is whether we want to identify cognition in this broad way with autopoiesis and biological autonomy or if we want to introduce a further restriction on cognition. Although I cannot conclusively discuss these questions in this paper, let me sketch a range of options.

### What Is the Mark of the Cognitive?

First of all, note that if we take intentionality to be equivalent to mental representation, then this implies that what the cell is doing counts as cognition. Whoever holds this view, must then answer the question what the cell (or certain elements of it) may represent. And indeed, such questions have been debated at length in discussions about naturalizing intentionality. Dretske (1986, p. 26), for example, introduced the case of marine bacteria which can only survive in the absence of oxygen and contain internal magnets (magnetosomes) which enable them to be directed toward oxygen-free waters (coinciding with geomagnetic north in the northern hemisphere and with geomagnetic south in the southern hemisphere). They basically avoid the surface. It is plausible to assume that this magnetic mechanism has evolved for this purpose, in the service of survival. These bacteria clearly exhibit *intentionality* in the sense of directedness, but it is much less clear that the magnetosome (or the bacterium) *represents* anything. Speculations about their representational content included “north” or “anaerobic water” (Millikan, 1989; Pietroski, 1992). Dretske (1986, p. 27) suggested that its natural meaning was “that there is relatively little oxygen in *that* direction” and that when a bacterium from the southern hemisphere were to be transplanted into the northern hemisphere, its magnetosome would effectively lead to its destruction, because it would lead it in the wrong direction. Dretske takes this to be “a plausible instance of misrepresentation.” But note that we are only forced to ponder such questions if intentionality is taken to be equivalent with representation. Although the bacterium is intentionally directed toward oxygen-free water (or however, *we* want to describe it^5^), and although it contains a mechanism that enables this (quite rigid, inflexible) directedness, this mechanism does not (need to) represent anything, especially because it does not allow for any flexibility in its behavior.^6^

If we reject the Equivalence Thesis, various other theoretical options remain. We are then in the position to allow that bacteria may exhibit basic intentionality based on their metabolic needs without thereby allowing this to involve representation. Still, although Enactivists of various stripes may agree concerning the separation of intentionality and representation, they may argue that we should consider this process in the single cell as a case of cognition. As mentioned above, Thompson (2007, p. 126) seems to be open to this idea when he holds that “cognition is behavior in relation to meaning and norms that the system itself enacts or brings forth on the basis of its autonomy.” Insofar as bacteria fulfill this criterion, they are capable of cognition. This option raises the question as to how useful it is *for us* to maintain such a broad notion of cognition since it has obvious consequences for cognitive science. If the mark of the cognitive is to exhibit intentional directedness in the basic biological sense of autopoiesis, then one consequence is that the subject matter of cognitive science is identical to the subject matter of biology. The study of cognitive systems then is the study of biological systems. To the extend to which we would like to learn more about how cognition works and what it is in *our* case, this makes sense. Yet, cognitive scientists may wish to delineate their field in a quite different way that is at the same time more restricted in one dimension and more flexible in another. Let me explain.

Thompson (2007, p. 128) holds the view that “the organizational properties distinctive of mind are an enriched version of those fundamental to life.” Yet, it is one thing to say that mind and life form a continuum, and quite another to hold that “mind is life,” that “the problem of mind is that of the problem of life” and that “where we discern life, we have everything we need to discern mind” (Noë, 2009, pp. 41–42). This latter view that slips from the “continuity” of life and mind (and thus, cognition) to the co-extensiveness of life and mind is quite useless for researchers in cognitive science who are considering (and developing) *artificial* systems and their *cognitive* capacities. After all, a strong reading of this option makes artificial cognitive systems impossible simply because they do not exhibit the right kind of biological setup. Since artificial intelligence has always been a goal of cognitive science, this view may strike many cognitive scientists as being way too restrictive. Di Paolo et al. (2017) prefer to use the more neutral notion of an “agent” and develop a hierarchy of notions of agency, starting with the minimal biological agency exhibited by bacteria, and culminating in the full-blown flexible and “open sensorimotor agency” of human beings and some other animals. With regard to the possibilities of artificial intelligence, they note that “the challenge for robotics is to create agency directly at the sensorimotor level” while bypassing the biological foundation found in organisms. “Insofar as the robot is capable of supporting the emergence, maintenance, and adaptive regulation of a network of *precarious* sensorimotor schemes it is a sensorimotor agent” (Di Paolo et al., 2017, p. 172). Yet, they hold that so far no robot meets this criterion since none of them can “self-individuate” so that they remain on the same level of behavioral flexibility like bacteria. I think that some kind of scale like the one Di Paolo et al. (2017) propose is needed and will be a welcome differentiation among the phenomena at issue. After all, “cognition” can also be seen as an umbrella term for a range of capacities that can be hierarchically ordered by complexity. Yet, note that the notions of agent and organism are quite distinct and pick out different sets of entities. On the one hand, the notion of an agent is much more flexible since it allows for the possibility of artificial agents that perform cognitive tasks, while the former restriction to organisms keeps its focus on the biological domain. On the other hand, one may worry that the ascription of something as an “agent,” being itself observer-relative and subject to taking the intentional stance toward any system of interest, will possibly include entities that are obviously non-cognitive.

Bourgine and Stewart (2004) and Bitbol and Luisi (2005) argue that autopoiesis may be sufficient for (or even constitutive of) life but that it is by itself insufficient for cognition. Continuity of mind and life (Thompson, 2007; Kirchhoff and Froese, 2017) does not imply that mind and life are co-extensive. But other philosophers are more inclusive, since they also consider the behavior of plants as falling within the realm of cognition. For example, in their defense of plant cognition, Garzon and Keijzer (2011) propose the criteria of *motility* and *sensorimotor organization* (first suggested by Jonas, 1966) as sufficient for minimal cognition. Incidentally, bacteria meet their criterion such that their proposal does not significantly differ from Autopoietic Enactivism as discussed above. They list striking instances of adaptive behavior in plants to do with movement, signal integration and other capacities, and argue that plants meet Jonas’ criteria once we differentiate between being free-moving on the one hand (like bacteria) and having self-induced motility on the other. Plants exhibit the latter despite not being free-moving, or so they argue. Jonas, by contrast, defended the stricter requirement that cognition demands free motility because only being free-moving allows for self-initiated action in the environment, as Garzon and Keijzer admit. It seems difficult to adjudicate this issue empirically given that we simply do not know enough yet about how many of these organisms, including bacteria, function. Much more work is needed here and evidence may decide the issue in one or the other direction.^7^

A way out of this dilemma is to abstract from the biological details and refer to the *organizational* or *functional* features of autopoietic systems. But note that this is to leave behind a stronger claim regarding the *identity* of life and mind by allowing quite different realizations of the very organization characteristic of life and mind. Once we focus on functions in living creatures, we have the choice among various candidates of which self-organization and self-maintenance are only two. This gives rise to the question what kind of functional architecture an organism must possess in order to meet the condition of being not only an “intentional system” but a “cognitive system.” A possible candidate may be the presence of a centralized control system that is responsible for (i.e., has the function of) processing information resulting from diverse inputs coming from multiple sources and putting this to use for executive control (Piccinini, unpublished). This is a way of differentiating the cognitive from the non-cognitive within the realm of (intentionally directed) organisms, since plants, fungi and bacteria may lack such an integrating control system. Although plants, bacteria and other organisms exhibit an autopoietic organization, their behavior then does not constitute a case of cognition because they lack the kind of complex processing and integration of information from various channels that is characteristic of cognition (on such a view).

Such a function of integrative control that is characteristic of cognitive processes may require a nervous system and brain. The underlying intuition here would be that nervous systems and brains provide the adequate architecture and functionality to instantiate mental representations, which in turn are considered as crucial for cognitive phenomena. Rejecting the Equivalence Thesis, this would situate the biological source of intentionality in the autopoietic organization of organisms, while restricting cognition to those processes involving mental representations which can – contingently – only be generated and sustained by nervous systems and brains. Rupert (2018) seems to have something like this in mind in his attempt to delineate “mere” representations from genuinely mental representations. According to his proposal, the latter must meet certain criteria one of which is that they occur in certain structures exhibiting a specific architecture.

This seems to be similar to the quite strict restriction of the realm of cognition that some philosophers prefer, e.g., by determining cognition as constituted by “particular kinds of processes involving non-derived representations” (Adams and Aizawa, 2001, p. 53). Adams and Aizawa propose this criterion in the context of the debate about whether cognitive processes can extend beyond the brain into the body and into the environment (Clark and Chalmers, 1998). Although this claim is silent about the “locus” of cognition, they argue that only certain kinds of mechanisms and processes can meet this criterion, namely, brainbound processes. Of course, this proposal is also not without problems. For one thing, it is controversial whether non-derived content exists (Dennett, 1987). Moreover, as Garzon and Keijzer (2011) point out, some researchers even consider “root-brains” (Baluška et al., 2004) in plants, which, despite being very different from “real brains,” are conceived as control centers regulating the plants’ adaptive behavior. This terminology suggests a certain liberty at work here and it does not only point to the need for conceptual clarifications of the kind proposed in this paper, but also to future empirical work that could decide some of these issues.

This (inconclusive) discussion of possible options further demonstrates the difficulty to go beyond certain terminological preferences, and arrive at the best delineation for cognitive science. What’s certain though is that the Representational Theory of Mind and Enactivism do by far not exhaust the range of theoretical options when it comes to determining the mark of the cognitive and the conceptual relations between cognitive, representational, and intentional phenomena. At bottom, the issue is whether cognitive scientists should favor a very broad notion of cognition or a more restricted one in order to investigate cognitive phenomena and it may well be up to the individual researchers to decide for themselves which notion is more useful to them. In the following last section, I would like to sum up and list some advantages of the general approach to a separation of intentionality and mental representation suggested here.

### Advantages

To recap, I have argued that intentionality can be naturalized if we take it primarily to be a feature of whole embodied agents, in our case, organisms, leaving room for the possibility of artificial intentional agents. This basic kind of biological intentionality is manifest even in a single cell’s autonomous self-production and maintenance of its identity via its dynamic interaction with the environment (see also Dennett, 1995, p. 205). The biological needs of the organism guide and control its intentional directedness and interaction with the environment, not the other way around. But this leaves us with the task of naturalizing mental representation which is conceived as a feature of mechanisms not whole organisms, a task not directly addressed in this paper. Of course, we could simply continue to *stipulate* that the notions of intentionality and mental representation can be used interchangeably, but we would loose the explanatory advantages that their clear separation offers. This separation allows, first and foremost, for a much more differentiated view of intentionality, representation, and mental representation, as indicated above. Focusing on “agents” of various complexities, Di Paolo et al. (2017) proposal has a similarly hierarchical taxonomy in mind. Such differentiations are especially important if we look at debates – in the context of Enactivism and Embodied Cognition – about the putative cognitive capacities of basic organisms, including cells, lower and higher animals and plants. It opens up the possibility, for example, that on the one hand, organisms can be directed at objects in various ways, the most basic of which do *not* involve mechanisms that carry representational content. But on the other hand, *having* such representational mechanisms enables an organism (or more general, a system) to be directed not only at ordinary objects or goals, but even at non-existent things via the (representational) capacity of imagination. In this way, the present framework can integrate what many philosophers take to be “Brentano’s problem,” namely the fact that intentionality involves the capacity to be directed at things that do not exist.

Moreover, a further advantage of this proposal is that it enables us to clearly situate predictive processing views and the impact of the free-energy principle (FEP) in discussions of cognition. Friston (2010) developed his predictive processing view of cognition and action as a special case of the tendency of organisms to resist entropy minimizing variational free energy, making the minimization of prediction error an instance of the FEP. The FEP originates in information theory to explain the self-organizing dynamics in systems that can remain in states far from equilibrium, and its application is thus quite independent from (or at least much more general than) mind and cognition (see also Friston, 2013). The proposed distinction underlying the conceptual move defended here considers the FEP, self-organizing dynamics and autopoiesis as pertaining to intentionality, whereas it considers the particular instance of the FEP, namely neuronal predictive processing, as pertaining to cognition and mental representation, providing an architecture sustaining representations. Thus, the proposed distinction avoids overly generous views according to which all systems that minimize free energy would have to be cognitive systems (see also Kirchhoff and Froese, 2017). In this way, the present proposal can integrate insights from the various frameworks distinguished above much better than Radical Enactivism. For example, it can much better make sense of Predictive Processing accounts of perception than Enactivism.^8^

Finally, the present conceptual move yields a layered model of intentionality with a phylogenetic as well as an ontogenetic dimension (Barresi and Moore, 1996; Schlicht, 2008). The central idea which is outside the scope of this programmatic paper is that there is not only a scale from simpler to complex creatures with an increasing range of intentional forms of directedness, but that also ontogenetically, our range of intentional forms of directedness unfolds during cognitive development. Taking this developmental stance enables the separation of non-representational kinds of intentionality from representational ones and the identification of a “distinctively *mental* kind of representation” (Morgan and Piccinini, 2017, p. 3) with the help of cognitive neuroscience within this broader framework of biological intentionality. As we have seen, Metzinger’s thematic focus is on mental representation that is also phenomenally experienced, a first-person phenomenon. Whether the “mental” realm is identical to the potentially “conscious” realm is an open issue, but the “cognitive” realm most plausibly goes way beyond and is in certain instances much more basic than that. In this sense, the foregoing considerations can be seen as a way of supporting and complementing Metzinger’s naturalization of phenomenal representation by providing a foundation of intentionality as such.

Intentionality enabled by mental representations allows for further differentiations from a developmental point of view: In their first year, young infants first engage only in dyadic relations with either caregivers or objects but already demonstrate an early understanding of the reciprocal nature of social interaction (Tronick et al., 1978). Only at around 9–12 months do they engage in triadic relations in scenes of joint attention (Eilan et al., 2005). According to Tomasello (1999), this indicates an understanding of others as *intentional agents* to be distinguished from the understanding of others as *mental agents*, which he associates with children’s understanding of false belief at 4 years (Wimmer and Perner, 1983). In-between though, children already start using their imagination and engage in *pretend play*, indicating that they do not only respond to a perceptual object or event, but also simultaneously ‘hold in mind’ a representation of an absent object or event. This capacity for counterfactual thinking is crucial for the development of social cognition (Gopnik and Wellman, 2012). At 18 months, children can complete a goal-directed action that an observed adult fails to complete (Meltzoff, 1995), indicating an understanding of the adult’s intentional relation to the task as well as the goal’s affordances. Various tasks show that children progress in their understanding of different mental states (Wellman et al., 2001), starting with understanding that people have divergent desires and diverse beliefs, via an understanding that they have access to knowledge, to the understanding of false belief and hidden emotion. Once worked out in detail, the present approach has the potential to yield a differentiated theory of intentionality, representation, and mental representation that (a) meets the constraint of providing a mark of cognition, (b) provides a naturalistic theory of intentionality, and (c) can incorporate insights from cognitive neuroscience and developmental psychology.

## Conclusion

This paper took off from Metzinger’s account of *phenomenal* representation in terms of his self-model theory. It emphasized the fact that he leaves unaddressed the project of providing a more general naturalistic theory of representation and intentionality. The paper then provided a sketch of how this could be developed using the means of a restricted version of Autopoietic Enactivism. Thus, the proposal is very much in spirit of Metzinger’s approach but attempts to support it by providing first steps toward a naturalistic theory of intentionality as such. The main conceptual move is to separate intentionality from representation – contra orthodox philosophy of mind and cognitive science – and conceive of intentionality as a feature of whole embodied agents and of representation as a feature of mental states (and their vehicles or underlying mechanisms, respectively). This proposal, defended in a similar fashion by Radical Enactivism, yields a division of labor in the sense that we need a naturalistic account of intentionality and another for mental representation while it is likely that they require completely different sets of explanatory tools. Space provided here only allowed attending to the first task. The upshot was that basic intentionality can be traced back to and explained by the autopoietic organization of organisms. Even single cells are intentionally directed to their environment based on their biological needs. That does not imply that they are thereby already cognitive systems or that this intentionality is cognitive or representational. Biological intentionality may be insufficient for cognition. Formulating stricter demands on cognition leaves open various options. The one suggested here is a functional restriction according to which cognition requires centralized control and integration of information coming from different sources. To the extend that this requires a physical mechanism like the brain and nervous system in organisms (and analogously, a physical mechanism of some other kind in an artificial system), it has the consequence that only creatures with such functional mechanisms can constitute an architecture sufficient for mental representation of the kind that Metzinger considers in his own account. Some of the advantages of this way of conceiving of intentionality and representation have been mentioned. The upshot is that intentionality is a developmental phenomenon that allows for various manifestations and varieties, both phylogenetically and ontogenetically. Rejecting the Equivalence Thesis thus provides us with a much more differentiated account of intentionality and representation than can be provided by theories based on the Equivalence Thesis. Yet, drawing this distinction does not imply Radical Enactivism but still leaves room for theoretical alternatives that accept mental representations in explanations of cognitive phenomena.

## Author Contributions

The author confirms being the sole contributor of this work and approved it for publication.

## Conflict of Interest Statement

The author declares that the research was conducted in the absence of any commercial or financial relationships that could be construed as a potential conflict of interest.

## Footnotes

1 Note that Crane (2003, p. 31) takes it that “the phrases ‘intentional inexistence,’ ‘relation to a content’ and ‘immanent objectivity,’ despite superficial differences between them, are all different ways of expressing the same idea: that mental phenomena involve representation or presentation of the world.”

2 Metzinger restricts the term “mental” for processes and events that can become conscious. Although this decision is more or less arbitrary, some terminological distinction is needed to single out “distinctively mental representations” from other kinds of representations. What is still needed is a rigorous terminology that defines how ‘representations,’ ‘mental representations,’ and ‘phenomenal representations’ are related and, most crucially, how they relate to the notion of ‘cognition.’

3 Up to this point, I have used the notions “agent” or “embodied agent” to denote the bearer of intentionality. As we will see in a bit, enactivists prefer to consider “organisms” as such bearers. Although certain organisms are embodied agents, it may be disputed that all organisms meet the criteria for being an agent. For that reason, it must be spelled out what we should take to be an agent. Also, not all embodied agents need be organisms. For example, from the start it was one of the chief goals of cognitive science to develop artificial intelligence, i.e., an artificial system that can perform cognitive tasks which have been considered as specifically human. We will discuss this issue further below.

4 Even though Enactivism comes in many flavors, the grounding of intentionality in autopoiesis, an idea that originates in the work of Maturana and Varela (1980), is widely accepted by the main supporters of Enactivism. Hutto and Myin (2013, p. 33) emphasize their “strong affinities with Autopoietic Enactivism,” while Noë (2009, p. 42) explicitly states that “mind is life.” Moreover, see Froese and Di Paolo (2011) and Kirchhoff and Froese (2017) who defend the mind-life continuity thesis as one of the main pillars of Enactivism.

5 One reviewer pointed out that if the bacterium’s mechanism was directed at *anaerobic water*, it would therefore also be directed toward *North* (and so on). Yes, we can provide many names for that toward which it is directed, but the question is whether we are forced to assume that it *represents* any of these contents. The suggestion is that it need not and that we need not assume that it does so for the purposes of explaining the bacterium’s behavior or the mechanism’s function within the bacterium.

6 Explaining the flexibility of behavior exhibited by certain agents has always been one important reason to posit mental representations in the first place (see Pylyshyn, 1984, chapter 2). Behavior being rigid and inflexible can thus be taken as an indication that positing mental representations that guide such behavior is unnecessary. I take the bacterium’s behavior to fall in this latter category. See also Di Paolo et al.’s (2017) recent distinctions among concepts of agency, ranging from the “minimal biological agency” exhibited by all organisms up to the “open sensorimotor agency” exhibited by humans and some higher animals whose behavior can “change in unpredictable, historically and culturally influenced manners” (Di Paolo et al., 2017, p. 171).

7 See Klumpp and Faivre (2016) for a review of the complexity of magnetotactic bacteria.

8 While Hutto and Myin only hint at a version of the predictive processing framework in radical enactive terms without alluding to “models” and “representations,” they do not develop this putative positive proposal anywhere (Hutto and Myin, 2017; Thompson, 2018).
